# Molecular Bases of Mechanisms Accounting for Drug Resistance in Gastric Adenocarcinoma

**DOI:** 10.3390/cancers12082116

**Published:** 2020-07-30

**Authors:** Jose J. G. Marin, Laura Perez-Silva, Rocio I. R. Macias, Maitane Asensio, Ana Peleteiro-Vigil, Anabel Sanchez-Martin, Candela Cives-Losada, Paula Sanchon-Sanchez, Beatriz Sanchez De Blas, Elisa Herraez, Oscar Briz, Elisa Lozano

**Affiliations:** 1 Experimental Hepatology and Drug Targeting (HEVEFARM), University of Salamanca, IBSAL, 37007 Salamanca, Spain; laurapsilva@usal.es (L.P.-S.); rociorm@usal.es (R.I.R.M.); masensio002@usal.es (M.A.); anapeleteiro@usal.es (A.P.-V.); anabelsanchez@usal.es (A.S.-M.); candelacives@usal.es (C.C.-L.); pausanchons@usal.es (P.S.-S.); beatrizsanchezbla@usal.es (B.S.D.B.); elisah@usal.es (E.H.); obriz@usal.es (O.B.); 2 Center for the Study of Liver and Gastrointestinal Diseases (CIBERehd), Carlos III National Institute of Health, 28029 Madrid, Spain

**Keywords:** apoptosis, chemoresistance, DNA repair, epithelial-mesenchymal transition, gastric cancer, metabolism, refractoriness, stomach, transport, tumor environment

## Abstract

Gastric adenocarcinoma (GAC) is the most common histological type of gastric cancer, the fifth according to the frequency and the third among the deadliest cancers. GAC high mortality is due to a combination of factors, such as silent evolution, late clinical presentation, underlying genetic heterogeneity, and effective mechanisms of chemoresistance (MOCs) that make the available antitumor drugs scarcely useful. MOCs include reduced drug uptake (MOC-1a), enhanced drug efflux (MOC-1b), low proportion of active agents in tumor cells due to impaired pro-drug activation or active drug inactivation (MOC-2), changes in molecular targets sensitive to anticancer drugs (MOC-3), enhanced ability of cancer cells to repair drug-induced DNA damage (MOC-4), decreased function of pro-apoptotic factors versus up-regulation of anti-apoptotic genes (MOC-5), changes in tumor cell microenvironment altering the response to anticancer agents (MOC-6), and phenotypic transformations, including epithelial-mesenchymal transition (EMT) and the appearance of stemness characteristics (MOC-7). This review summarizes updated information regarding the molecular bases accounting for these mechanisms and their impact on the lack of clinical response to the pharmacological treatment currently used in GAC. This knowledge is required to identify novel biomarkers to predict treatment failure and druggable targets, and to develop sensitizing strategies to overcome drug refractoriness in GAC.

## 1. Introduction

Multidrug resistance (MDR) phenotype is a common trait of many solid tumors and blood malignancies, which can be present before starting any pharmacological treatment (primary or intrinsic resistance) or can be developed or potentiated in response to anticancer drugs (secondary or acquired resistance). There is a variety of mechanisms of chemoresistance (MOCs), several of which are simultaneously present in the tumor and commonly act in a synergistic manner. The consequence is the appearance of cross-resistance to different antitumor drugs, which markedly limits the options of pharmacological treatment and hence the positive outcome of the patients. This situation is particularly harmful when it affects types of cancer, like gastric adenocarcinoma (GAC), that are often diagnosed in an advanced stage, when surgical removal is not recommended. Hence, the patient outcome only relies on the success of pharmacological treatment. GAC, the most common histological type of gastric cancer, is the fifth most frequent cancer, but the third among the deadliest cancers [1,2]. In addition to marked chemoresistance, its high mortality is due to a combination of other factors, such as silent evolution, late clinical presentation, and underlying genetic heterogeneity.

Although there is no single pharmacological regimen established for the treatment of metastatic or advanced unresectable GAC, the most commonly used drugs as first-line therapy are platinum derivatives (cisplatin, oxaliplatin), 5-fluorouracil (5-FU) and other pyrimidine analogs (capecitabine) and anthracyclines (doxorubicin, epirubicin), either administered as single agents or frequently in combination among them. More recently, trastuzumab, a monoclonal antibody that targets the epidermal growth factor receptor 2 (HER2), has been included in the treatment of choice for HER2-positive GAC. As second-line chemotherapy, taxanes (paclitaxel, docetaxel), camptothecins (irinotecan), and ramucirumab, a targeted therapy against angiogenesis, have been used [3]. However, the marked MDR phenotype of GAC makes the available chemotherapy scarcely effective.

**Figure 1:**
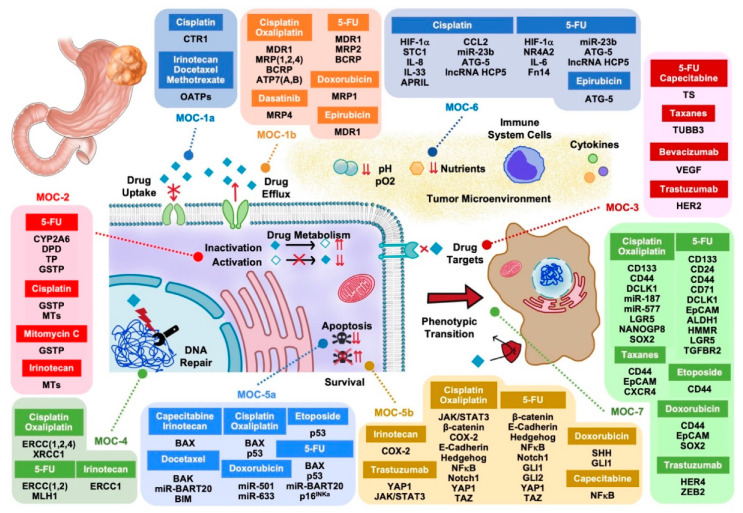
Proteins and non-coding RNAs accounting for drug resistance in gastric adenocarcinoma. MOC, mechanism of chemoresistance.

In this review, we have summarized the updated knowledge regarding the molecular bases accounting for the lack of clinical response of GAC to chemotherapy, using the previously proposed classification into seven groups of MOCs [4,5]. This division has been established based on whether the mechanisms cause reduced drug uptake (MOC-1a), enhanced drug efflux (MOC-1b), a low proportion of active agents in tumor cells due to impaired pro-drug activation or active drug inactivation (MOC-2), changes in molecular targets sensitive to anticancer drugs (MOC-3), enhanced ability of cancer cells to repair drug-induced DNA damage (MOC-4), decreased function of pro-apoptotic factors *versus* up-regulation of anti-apoptotic genes (MOC-5), changes in tumor cell microenvironment reducing the efficacy of antitumor agents (MOC-6) and phenotypic transformations, including epithelial-mesenchymal transition (EMT) and the appearance of stemness characteristics (MOC-7) (Figure 1).

## 2. Mechanisms of Chemoresistance Type 1 (MOC-1)

Reduced drug uptake (MOC-1a) or enhanced drug export (MOC-1b), which are dependent on changes in the expression levels or the presence of genetic variants affecting proteins that constitute the “transportome”, may determine low intracellular concentrations of antitumor drugs and hence the lack of GAC response to pharmacological treatments (Table 1) [6,7].

### 2.1. Uptake Transporters (MOC-1a)

Several plasma membrane transporters belonging to the Solute Carrier (SLC) superfamily of proteins play a critical role in drug uptake. Most of these carriers are weakly expressed in GAC [8,9]. Exceptions include the copper transporter CTR1 (*SLC31A1* gene), whose presence may determine the sensitivity to cisplatin [9,10]. Equilibrative nucleoside transporters (ENTs, *SLC29* family) participate in the uptake of pyrimidine analogs. As the expression of ENT1 in 5-FU-resistant GAC cells is high, other mechanisms different from reduced ENT1-mediated uptake must account for this phenotype [11]. Regarding organic anion-transporting polypeptides (OATPs, *SLCO* family), there is a higher abundance of *SLCO1B3* mRNA in GAC than in paired adjacent non-tumor tissue [9], which has been confirmed in cultured GAC cells [12]. Microarray analysis revealed a higher expression of several OATP isoforms (2B1, 3A1, 4A1, and 5A1) in GAC biopsies than in healthy gastric tissue [12]. Because the uptake of some drugs used in the second-line treatment of GAC, such as irinotecan, docetaxel, and methotrexate, can occur through OATPs, their expression may determine the sensitivity of GAC to these drugs. Interestingly, the splice variant of *SLCO1B3* known as cancer-type OATP1B3 is highly expressed in GAC, whereas it is absent in the healthy stomach. However, the functional repercussion of this variant remains controversial [9].

### 2.2. Export Pumps (MOC-1b)

Owing to their ability to export a large variety of antitumor drugs from cancer cells, which reduces their pharmacological effect, ATP-binding cassette (ABC) transporters are commonly involved in the MDR phenotype of GAC. The prototypic ABC pump is multidrug resistance protein 1 (MDR1 or P-glycoprotein, *ABCB1*), which is involved in the chemoresistance of many tumors. However, its relevance in GAC remains uncertain. Low or moderate *ABCB1* mRNA levels in GAC biopsies have been reported [9]. In addition, immunohistochemical analysis revealed that the protein is predominantly localized intracellularly [13], where it cannot carry out its drug export function. This is not in agreement with reports suggesting a role of this pump in GAC chemoresistance. Thus, although platinum derivatives are not MDR1 substrates, high expression of *ABCB1* has been reported in GAC biopsies from patients classified as poor responders to platinum-based therapy [14], while in vitro assays have demonstrated a relationship between MDR1 expression in GAC and cisplatin [15] and oxaliplatin [16] resistance. In GAC clinical samples, a positive correlation between MDR1 and dCTP pyrophosphatase 1 (DCTPP1) expression has been detected [17]. This is consistent with the fact that DCTPP1 is associated with reduced methylation of the *ABCB1* promoter. Accordingly, low DCTPP1 expression is accompanied by a higher degree of methylation in this DNA region, which reduces MDR1 expression. Interestingly, in GAC cells, knock-down of DCTPP1 resulted in MDR1 down-regulation together with enhanced sensitivity to 5-FU, even though this drug is not an MDR1 substrate either [17]. In vitro assays also support a relevant role of MDR1 in GAC response to epirubicin. Thus, *ABCB1* knock-down in an epirubicin-resistant GAC cell subline decreased the efflux of this drug and increased its cytotoxicity [18]. In addition, pharmacological treatment of GAC cells has been reported to modulate MDR1 expression and therefore cell sensitivity to other drugs. For example, sorafenib can reverse cisplatin resistance in GAC cells through down-regulation of MDR1 expression [19]. Besides, tamoxifen can reverse the MDR phenotype by enhancing the sensitivity to cisplatin, 5-FU, and doxorubicin through a reduction in MDR1-mediated drug efflux [20,21].

MRP1 (*ABCC1*) is highly expressed in GAC tissues [9]. MRP1 expression has been proposed as a marker for chemoresistance in GAC, particularly associated with acquired cisplatin resistance. Thus, enhanced MRP1 expression in GAC cells obtained from naïve patients after cisplatin treatment and in the cisplatin-resistant GAC cell line KATOIII/DDP has been found [22]. Indirect evidence further supports that MRP1 is involved in cisplatin resistance [23,24]. MRP1 overexpression in vitro reduced the cytotoxic effect of doxorubicin, whereas tanshinone IIA (an abietane diterpene) potentiated doxorubicin effect, even in resistant GAC cells, by MRP1 inhibition [25].

MRP2 (*ABCC2*) is not abundantly expressed in GAC [9]. The SNP located in the 5ʹUTR mRNA (c.-24C>T, rs717620) can be involved in modulating *ABCC2* expression, and hence affect drug effectiveness. Indeed, patients with CC genotype had a worse response to oxaliplatin and fluoropyrimidine-based treatment than those with TT and TC genotypes [26].

MRP4 (*ABCC4*) is highly expressed in GAC tissues [9] and also in cisplatin-resistant GAC cell lines (e.g., SGC7901/DDP), in which inhibition of MRP4 expression by siRNA reversed cisplatin resistance [27]. MRP4 has also been associated with a lower response of GAC to other drugs, such as dasatinib [28].

Breast cancer resistance protein (BCRP, *ABCG2*) also confers GAC cells resistance to cisplatin [29], whereas BCRP inhibition using genetic manipulation or inhibitors, such as fumitremorgin C, sensitizes them to this drug [30]. These findings were consistent with results obtained using xenograft mice models [30]. Furthermore, BCRP expression has been associated with poor overall survival (OS) of GAC patients who underwent cisplatin-based therapy [30], and with a higher incidence of relapse in patients treated with 5-FU [31]. These findings have fostered the development of strategies to overcome BCRP-mediated drug resistance. For example, ribozymes have been used to reduce *ABCG2* mRNA levels and hence to overcome BCRP-mediated drug resistance. This strategy results in enhanced sensitivity of GAC cells to antitumor drugs that are BCRP substrates [32].

Apart from ABC pumps, Menkes and Wilson proteins (*ATP7A* and *ATP7B* genes, respectively) are ATP-dependent copper transporters with an uncertain role in GAC chemoresistance. In oxaliplatin resistant GAC cells, *ATP7A* expression was increased [33]. Moreover, *ATP7B* is highly expressed in GAC, being significantly higher in poorly-differentiated to undifferentiated tumors than in moderately- to well-differentiated ones. Although some in vitro studies have suggested a link between *ATP7B* expression and cisplatin resistance [34], whether this fact affects the pharmacological treatment of GAC is yet unknown.

## 3. Mechanisms of Chemoresistance Type 2 (MOC-2)

GAC cells are often able to inactivate antitumor drugs or to reduce pro-drugs activation due to changes in the expression or activity of metabolic enzymes involved in detoxification, leading to a lower intracellular amount of active agents (Table 2) [6,35].

Cytochrome P450 (CYP) is an important group of enzymes responsible for the metabolism of many anticancer drugs, but also for activating many pro-drugs. For instance, tegafur, included in first-line chemotherapy of GAC, is converted into 5-FU by CYP2A6 [36]. This enzyme is encoded by a highly polymorphic gene with over 50 SNPs, some of which reduce or abolish its activity. These genetic variations affect tumor sensitivity to 5-FU and, hence, shorten the survival rates of treated patients [37,38,39,40].

Thymidine phosphorylase (TP) is crucial in the first step of metabolic biotransformation of 5-FU into the active metabolite fluorodeoxyuridine monophosphate. TP expression has been directly related to the sensitivity to 5-FU. However, results are controversial for GAC, as some authors have reported a correlation between low TP levels, development of 5-FU resistance and poor prognosis of patients [41,42]; whereas others have not observed such a relationship [43,44] or even have found high TP levels associated with worse survival, probably due to its role in angiogenesis, cancer invasiveness and metastasis [45].

Carboxylesterases (CES) catalyze the hydrolysis of a wide variety of compounds, including carbamates, esters, thioesters, and amides. In GAC, high CES2 activity has been associated with an enhanced effect of capecitabine, which is converted into 5-FU by this enzyme in tumor cells [53]. CES2 is also involved in the intracellular activation of irinotecan, another pro-drug used as second-line chemotherapy in GAC that must be biotransformed into its active metabolite SN-38 [54]. There is a weak CES2 expression in healthy gastric tissue, however, a marked overexpression in GAC has been observed mainly when cancer appears associated with Barrett’s esophagus [55]. Accordingly, CES2 has been proposed as a predictive biomarker of irinotecan response in this group of GAC patients [55].

The overexpression of dihydropyrimidine dehydrogenase (DPD), the main enzyme in 5-FU catabolism, induces lack of sensitivity to this drug. Thus, DPD mRNA levels have been proposed to predict a worse response to 5-FU derivatives in GAC patients [47,48].

Metallothioneins (MTs) are small cysteine-rich proteins involved in the mechanisms of protection against the toxicity of many xenobiotics. The relationship between MTs and the response to chemotherapy in GAC patients is controversial. Thus, the refractoriness to irinotecan and cisplatin may be due in part to MTs because (i) MTs have been found up-regulated in GAC, affecting the efficacy of cisplatin and irinotecan [51,52]; (ii) irinotecan induces MTs up-regulation promoting the development of chemoresistance in GAC patients [51]; (iii) the combination of MTs overexpression and p27 down-regulation seems to be related with poor prognosis of GAC patients [56]. In contrast, another study reported that GAC with a higher MT2A expression showed a better response to chemotherapy and prolonged survival [57,58]. Moreover, some studies revealed lower MTs expression in GAC than in the surrounding healthy tissue [59].

Three major glutathione-S-transferase (GST) isoenzymes, i.e., GSTM (mu) 1, GSTT (theta) 1, and GSTP (pi) 1, widely expressed throughout the human gastrointestinal tract [60], may be responsible for enhanced resistance to several anticancer drugs [61]. Among them are platinum derivatives (cisplatin, oxaliplatin, and carboplatin) [49,50], whose conjugation with glutathione results in the formation of inactive adducts. Thus, GSTP may play a significant role in the early resistance of GAC as its overexpression has been correlated to both intrinsic and acquired resistance to 5-FU, cisplatin, and mitomycin C in GAC [49,50]. Moreover, in patients with advanced GAC, the variant GSTP1*B (c.313A>G in exon 5; p.lle105Val), associated with lower enzymatic activity, has been related to a better prognosis and response to the oxaliplatin- and 5-FU-based regimen as first-line treatment [62].

UDP-glucuronosyltransferases (*UGT* genes) play essential roles in the metabolism of xenobiotics. Members of the UGT1A family, suggested to be involved in gastric carcinogenesis, are differentially expressed in GAC [63]. Unlike UGT1A3 and UGT1A5, which are down-regulated in GAC, UGT1A6 expression is higher in GAC than in healthy tissue [63]. Moreover, polymorphisms in *UGT1A1*, considered a crucial enzyme in irinotecan metabolism, have been associated with different clinical outcomes of patients with advanced GAC treated with this drug [64].

## 4. Mechanisms of Chemoresistance Type 3 (MOC-3)

The appearance of changes in the expression and function of molecular targets can help GAC scaping from anticancer agents [4] (Table 3). This is the case, for example, of 5-FU, whose mechanism of action is mediated by the inhibition of thymidylate synthase (TS), which plays a crucial role in DNA synthesis. There are conflicting data on the usefulness of determining TS expression to predict clinical outcome of GAC patients treated with 5-FU, as some studies did not find a relationship between them [65,66], while others reported that high TS expression, determined by immunohistochemistry, was predictive of worse outcomes of patients receiving adjuvant oxaliplatin plus the 5-FU precursor capecitabine [67]. In addition, a meta-analysis using 20 studies identified several TS polymorphisms associated with clinical outcomes of GAC patients treated with platinum/5-FU-based chemotherapy. Thus, 2R/2R and 2R/3R genotypes (corresponding with a double or triple repeat of a tandem sequence in the TS promoter—TS enhancer region or TSER—) were associated with shorter OS [68].

DNA topoisomerases (TOPO I and II) are the molecular targets of drugs used in combined regimes for GAC, such as irinotecan (TOPO I inhibitor), doxorubicin, and epirubicin (TOPO II inhibitors). No relationship between TOPO I expression and the response to irinotecan and docetaxel has been reported [69]. In contrast, TOPO II expression was significantly lower in GAC cells isolated from fresh specimens that were resistant to doxorubicin, but also to hydroxycamptothecin and mitomycin C, which are not associated with TOPO II [50].

Docetaxel and paclitaxel target both α- and β-tubulin subunits, which stabilizes microtubules and subsequently blocks cell cycle progression. Immunohistochemical analysis revealed that β-tubulin-III (*TUBB3*) expression was higher in GAC than in benign gastric mucosa lesions [70], which had been associated with resistance to docetaxel-based chemotherapy [71]. Moreover, immunohistochemistry of β-tubulin-III was proposed to predict the response to taxane-based chemotherapy in recurrent and metastatic GAC patients [72]. A relationship between *TUBB3* mRNA levels and clinical outcomes in patients with advanced GAC receiving palliative treatment with docetaxel, cisplatin, and 5-FU has also been found [66]. This was consistent with the findings that high *TUBB3* expression and microtubule-associated protein tau (*MAPT*) inversely correlated with the sensitivity to paclitaxel in cells isolated from fresh tumor tissue [73].

Receptors with tyrosine kinase activity play a pivotal role in signal transduction and constitute the targets for tyrosine kinase inhibitors (TKIs). The overexpression of epidermal growth factor receptor (EGFR) in a high number of GACs [74] pointed at this protein as a promising target in this type of cancer. However, several trials have shown no increase in OS by including anti-EGFR antibodies, such as cetuximab (EXPAND trial) or panitumumab (REAL-3 trial), in the treatment of unselected patients with advanced esophagogastric adenocarcinoma [75,76]. Moreover, no better response was observed by combining panitumumab with perioperative chemotherapy (NEOPECX trial) [77].

Trastuzumab, ramucirumab, bevacizumab, and apatinib are targeted agents used in the treatment of GAC. Trastuzumab is a monoclonal antibody that interferes with HER2 and that, combined with conventional chemotherapy, is the treatment of choice for HER2-positive GAC. The ToGA trial demonstrated that this treatment improved OS without adverse effects in patients with advanced GAC, whereas in patients with low HER2 expression, the beneficial effect was milder [78,79]. In addition, intrinsic and acquired resistance to trastuzumab has been inversely correlated with HER2 copy number in GAC [80]. Ramucirumab is an antibody that inhibits vascular endothelial growth factor receptor 2 (VEGFR-2) and has shown some benefits in GAC patients, both alone and in combination with paclitaxel [81,82]. High VEGFR-2 endothelial expression was associated with a non-significant prognostic trend toward shorter progression-free survival (PFS) [81]. Another inhibitor of VEGFR-2, apatinib, is being tested in clinical trials [82]. A recent meta-analysis has shown that this drug was the best among assayed targeted therapies in improving OS, PFS, and objective response rate, both alone and in combination with conventional chemotherapy [83]. In the latter case, the beneficial effect has been associated with the ability of apatinib to reverse MDR1 and BCRP transport function [84]. Bevacizumab is an antibody that binds to the vascular endothelial growth factor (VEGF), blocking the interaction with its receptors. Low VEGF expression has been associated with worse clinical outcome in patients with advanced GAC treated with bevacizumab [85]. A meta-analysis of individual patient data found that variants in the VEGF pathway, including VEGF-A and VEGF-C, have potential value in predicting bevacizumab treatment outcome across tumor types [86]. However, these results need to be validated in larger cohorts of GAC patients.

## 5. Mechanisms of Chemoresistance Type 4 (MOC-4)

The dynamic balance between DNA damage and repair depends on the type of injury and the activity of a variety of repair mechanisms that preserve genome integrity, such as nucleotide-excision repair (NER), base-excision repair (BER), mismatch repair (MMR), non-homologous end-joining (NHEJ) and homologous recombination (HR) systems. Aberrant over-activation of DNA repair mechanisms (MOC-4) could prevent tumor cells from drug-induced apoptosis and, therefore, it may play a pivotal role in GAC chemoresistance (Table 4).

NER system can repair DNA adducts and crosslinks caused by alkylating agents like cisplatin. More than 30 factors work together in this complex process, among which the excision repair cross-complementing proteins (ERCC) and the product of the *Xeroderma pigmentosum* (XP) group genes stand out. Alterations in several NER proteins have been related to the effectiveness of GAC treatments, especially those based on platinum-derived drugs [87]. In this regard, one of the most studied NER enzymes is ERCC1. Immunohistochemical analysis revealed that ≈70% of advanced GAC samples presented positive ERCC1 staining, which was associated with lower OS and time-to-progression (TTP) in patients treated with neoadjuvant FOLFOX regimen (leucovorin/5-FU/oxaliplatin) [65]. A meta-analysis that included more than 1400 patients confirmed that high ERCC1 expression inversely correlated with the response to platinum-based chemotherapy, especially in Asian patients [88]. Moreover, high ERCC1 levels were associated with a lower response rate in GAC patients treated with irinotecan plus cisplatin [89]. In GAC, changes in ERCC1 expression have been associated with miR-122 and miR-139-5p, whose expression is reduced in cisplatin-resistant cells and inversely correlated with that of ERCC1. When both miRNAs were induced in vitro, ERCC1 protein levels decreased, and the sensitivity to cisplatin was restored [90,91].

Besides expression levels, ERCC1 genetic variants can also influence the pharmacological sensitivity of GAC to DNA-damaging agents. The rs11615 polymorphism has been associated with an unsatisfactory response and shorter OS after 5-FU- [92] and oxaliplatin-based [93] chemotherapy. However, other studies could not confirm these findings [94,95]. There is also controversy regarding the prognostic value of rs3212986, a mutation that affects the 3′UTR region of *ERCC1* mRNA. Whereas, some studies associate the presence of the rs3212986 variant with the outcome of patients receiving cisplatin-based treatment [96] and the FOLFOX regimen [94], a meta-analysis involving 11 Chinese cohorts could not establish any relationship between rs3212986 and chemotherapy sensitivity in GAC [97].

Regarding other NER proteins, overexpression of ERCC2 (or XPD), either alone or combined with high ERCC1 levels, has been associated with poor OS and therapy response in GAC patients treated with neoadjuvant FOLFOX [98]. ERCC4 (or XPF), which forms a heterodimer with ERCC1 to repair DNA, has also been associated with GAC chemoresistance. In vitro studies have suggested that ERCC4 expression could be modulated by several miRNAs, inducing cisplatin resistance in GAC cells [91,99]. However, the few clinical studies performed up to date have not found a link between ERCC4 expression [45] or its genetic variants [104] with the sensitivity to platinum-containing drugs.

XRCC1, an important component of BER mechanism, can also confer resistance to cisplatin. BER corrects small base lesions that do not distort the DNA helix structure and XRCC1 serves as a scaffolding protein that interacts with other enzymes that repair DNA. Increased XRCC1 expression was found in cisplatin-resistant GAC cells and down-regulation of XRCC1 by the proteasome cofactor TXNL1 restored sensitivity to this drug [100]. Concerning genetic variants, an association between XRCC1 rs25487 and the clinical outcome of GAC patients treated with oxaliplatin has been found [101].

MMR is a strand-specific mechanism that recognizes and repairs mismatched bases and insertions or deletions. Deficiencies in the MMR machinery may favor the generation of genetic mutations in tumor cells that lead to the so-called “microsatellite instability (MSI) phenotype”, which appears in 15–30% of GAC [105]. MSI has been associated with decreased expression of several MMR proteins (MLH1, PMS2, and PMS1) in GAC [106]. Interestingly, in terms of improved disease-free survival (DFS), GAC patients with low MSI status were more sensitive to 5-FU-based adjuvant chemotherapy than those with high MSI [103]. Moreover, the lack of MLH1 expression has been associated with chemoresistance in patients treated with neoadjuvant 5-FU-based chemotherapy [102], probably due to an enhanced MSI phenotype of GAC.

Reduced expression of BRCA1 and BRCA2, members of the HR repair system, has been found in approximately 17% of GAC patients who had received postoperative adjuvant chemotherapy; however, this down-regulation did not correlate with any clinical parameter [107]. Instead, the BRCA1 polymorphism rs799917 may have a positive impact on the OS of patients treated with a taxane (docetaxel or paclitaxel) and cisplatin-based therapies [108].

## 6. Mechanisms of Chemoresistance Type 5 (MOC-5)

### 6.1. Pro-Apoptotic Factors (MOC-5a)

The impaired function of pro-apoptotic proteins often results in an insufficient pharmacological response of GAC (Table 5). An essential player in drug-induced apoptosis is p53 that, in response to cell stress, can arrest proliferation and promote cell death. However, p53 function is commonly abolished in GAC due to the loss of heterozygosity and the presence of loss-of-function and dominant-negative mutations in the *TP53* gene [109]. The rs1042522 variant (p.Arg72Pro), in addition to be associated with a higher risk of GAC development [110], is also relevant in the response to chemotherapy. Therefore, the presence of the rs1042522 variant is considered an independent prognostic factor for a worse response to 5-FU plus paclitaxel [111] and cisplatin-based chemotherapy in GAC [112]. Regarding the relationship between p53 expression and chemoresistance in GAC, conflicting results have been reported. While decreased p53 expression has been correlated with GAC refractoriness to neoadjuvant therapy with 5-FU and cisplatin [107] and to preoperative high dose chemotherapy based on etoposide, cisplatin, and mitomycin C [106], other studies have found a higher response rate to chemotherapy in patients with p53 negative tumors [113]. Given that controversy, Xu et al. [114] performed a meta-analysis comprising thirteen published studies and concluded that p53 positive status (high expression of p53 protein, regardless of the presence of *TP53* mutations) is associated with better response to neoadjuvant chemotherapy. Therefore, it has been proposed that p53 status could be a predictive marker for the response to chemotherapy in GAC [114]. On the other hand, some GAC patients have gain-of-function mutations in *TP53*, mainly affecting Arg175, Gly245, Arg248, Arg273, and Arg282 residues, that lead to the acquisition of novel oncogenic properties promoting tumor growth and progression. The presence of these mutations is related to enhanced *HER2* expression, leading to overactivation of HER2-mediated survival pathway [115], and has been associated with a worse OS and PFS in GAC patients [116,117].

*CDKN2A* gene encodes the p16^INKa^ protein, which participates in the regulation of cell cycle and apoptosis activation. The loss of p16^INKa^ expression due to the hypermethylation of *CDKN2A* promoter occurs in approximately half of GAC tumors [118] and is especially prevalent in Epstein-Barr virus-associated GAC [119]. This has been associated with a worse response of GAC to adjuvant 5-FU therapy [120]. However, other studies have identified the hypermethylation of the *CDKN2A* promoter as a possible predictor of longer PFS in these patients [121].

Dysregulation of the intrinsic or mitochondrial pathway of apoptosis activation also contributes to GAC chemoresistance [122]. Low BAX expression has been associated with reduced response in patients who had received 5-FU plus cisplatin [123], COI (capecitabine, oxaliplatin plus irinotecan) or FOLFOX [124,125]. BAK down-regulation enhances the resistance to docetaxel-induced apoptosis in GAC cell lines [126]. In addition, in patients with low BAK expression, a worse response to chemotherapy regimes containing docetaxel has also been reported [126]. Pro-apoptotic BH3-only proteins, such as BAD, BIM, and BID, directly activate BAX and BAK and inhibit anti-apoptotic factors of the intrinsic apoptotic pathway. Changes in the expression of these proteins can favor GAC chemoresistance. Reduced BIM expression has been associated with shorter OS in docetaxel-treated patients [127]. Moreover, miR-BART20-5p-mediated BAD down-regulation in GAC cells increases their resistance to docetaxel- and 5-FU-induced apoptosis [128]. The miR-501-mediated down-regulation of BLID, another member of the intrinsic pathway, promotes doxorubicin resistance through inactivation of caspases 3 and 9, and phosphorylation of AKT [129].

On the other hand, the impairment of elements involved in the extrinsic pathway of apoptosis activation also participates in GAC chemoresistance [130]. For instance, the loss of FADD, which transmits the signal from cell-death receptors to procaspases, is a frequent event in GAC [131]. Moreover, the overexpression of miR-633 increases doxorubicin resistance by targeting FADD [132].

### 6.2. Survival Pathways (MOC-5b)

Through apoptosis inhibition, dysregulation of survival mechanisms, such as NF-ĸB, Hedgehog and Notch pathways, can also drive resistance to chemotherapy in GAC (MOC-5b). Aberrant NF-ĸB pathway hyperactivation, a common feature in GAC [133], has been associated with resistance to cisplatin in vitro [14]. Antitumor drug-induced cellular stress activates this pathway in GAC cells, which favors their survival and the appearance of acquired chemoresistance [134]. As a consequence of NF-ĸB dysregulation, anti-apoptotic factors, such as survivin, BCL-XL, and XIAP are up-regulated [135]. Thus, survivin serum levels have been proposed as a predictor of clinical response to the modified DCF (docetaxel, cisplatin, and 5-fluorouracil) regimen in advanced GAC [136].

The Wnt/β-catenin pathway is hyperactive in most GACs [149], which has been associated with unsatisfactory clinical outcome [147]. *Helicobacter pylori* infection contributes significantly to this pathway dysregulation. CagA is a virulence factor of *H. pylori* that can induce β-catenin accumulation in cytoplasm and nucleus and Wnt/β-catenin-dependent expression of SOX9, NANOG, and OCT4 [147]. The loss of E-cadherin, which increases β-catenin levels, is more frequent in chemoresistant than in chemosensitive GACs [141]. In addition, several mutations in *CTNNB1* (encoding β-catenin), *APC*, and *FBXW7* have been associated with a lower OS and shorter PFS of GAC patients treated with first-line chemotherapy [139].

Regarding the Hedgehog pathway, its hyperactivation in GAC has also been related to a more aggressive and chemoresistant phenotype [150]. Tissue damage caused by chronic *H. pylori* infection is one of the factors that activate the Hedgehog pathway [142]. Exposure of GAC cells to 5-FU also up-regulated the target genes *GLI1* and *GLI2* [31], whose overexpression has been associated with a high incidence of relapses in patients treated with 5-FU [31]. The overexpression of the ligand SHH has been linked to resistance to doxorubicin-induced apoptosis [146].

Aberrant activation of Notch signaling is also involved in GAC chemoresistance [151]. High expression of Notch 1 receptor has been found in GAC patients who do not respond to neoadjuvant 5-FU and cisplatin [144]. Interestingly, in cisplatin-resistant GAC cells, Notch 1 up-regulation, through the long non-coding RNA (lncRNA) AK022798, promoted MDR1 and MRP1 expression, together with decreased caspase-3 and caspase-8 levels [152]. The Notch pathway regulates the expression of cyclooxygenase-2 (COX-2), an inducible enzyme expressed in the gastric mucosa during inflammation and carcinogenesis, which is related to the sensitivity of patient-derived GAC cells to antitumor drugs [140].

Dysregulation of the Hippo pathway leads to the oncogenic accumulation of YAP1, and TAZ in the nucleus and it has been associated with GAC chemoresistance [153]. High expression of both proteins has been correlated to a decreased sensitivity in vitro [148], and a less satisfactory outcome in patients with advanced GAC treated with adjuvant chemotherapy [139]. Increased activity of PI3K/AKT and JAK/STAT3 pathways has also been related to a lower response of these patients to cisplatin [143] or trastuzumab [145].

## 7. Mechanisms of Chemoresistance Type 6 (MOC-6)

Tumor cells interact with their surrounding microenvironment, which comprises tumor stroma, blood vessels, recruited inflammatory cells, and other several types of associated cells. They generate factors that affect tumor progression and dramatically alter the response to chemotherapy [154]. Hypoxia is one of the common characteristics of the tumor microenvironment. This has been associated with enhanced resistance to chemotherapy in GAC through a mechanism that involves hypoxia-inducible factor-1 (HIF-1) expression (Table 6). HIF-1 regulates several cellular processes, including metabolism and vascular homeostasis, and affects the expression of genes involved in drug resistance, such as MDR1 and MRP1 pumps and the apoptosis inhibitor BCL-2 [155]. In GAC, HIF-1α induces resistance to platinum derivatives by preventing apoptosis through dysregulating the expression of miR-27a and miR-421 [156,157], and the lncRNA PVT1 [158]. In vitro studies have shown that hypoxia-related 5-FU and cisplatin resistance was mediated by inhibition of p53 and activation of NF-κB [159]. The sensitivity of GAC cells to 5-FU and oxaliplatin was enhanced by silencing HIF-1α [160]. Moreover, HIF-1α expression was associated with relapse in GAC patients treated with adjuvant 5-FU after surgery [161].

The expression of stanniocalcin-1 (STC1), a glycoprotein involved in calcium/phosphate homeostasis, is enhanced in hypoxic conditions and promotes tumor cell invasion and resistance to cisplatin. Thus, STC1 overexpression in GAC cells in vitro inhibited apoptosis by up-regulation of BCL-2 and decrease in cleaved-caspases-3/9 levels and altered cell metabolism by down-regulating cytochrome c [162].

Another crucial characteristic of the tumor microenvironment that favors chemoresistance is the presence of inflammation. In GAC, this is caused by stress-inducing conditions, host immune response, and chronic infection with *H. pylori*. The orphan nuclear receptor 4A2 (NR4A2) is induced by prostaglandin E2 that is released under inflammatory conditions. NR4A2 inhibits apoptosis and activates the promoter of osteopontin, an inflammatory mediator that affects tumor progression and angiogenesis. High NR4A2 expression in GAC cells conferred resistance to 5-FU by preventing drug-induced apoptosis. In addition, the detection by immunohistochemistry of high NR4A2 expression in tumor tissue was associated with worse survival rates in patients receiving post-operative 5-FU-based chemotherapy [163].

Cancer-associated fibroblasts (CAFs), which are part of the tumor microenvironment, interact with tumor cells through several secreted signals. In particular, cytokines such as IL-6, IL-8, and IL-11, which contribute to inflammation, have been associated with chemoresistance development in GAC. Thus, using different experimental models and a specific monoclonal antibody against the IL-6 receptor, it was demonstrated that IL-6 inhibited 5-FU-induced apoptosis. Moreover, clinical data suggested that IL-6 up-regulation correlated with a more unsatisfactory response to 5-FU in GAC patients [167]. Furthermore, IL-8 can activate NF-κB and up-regulate *ABCB1*, causing cisplatin resistance in GAC cells [14]. Similar results were observed for oxaliplatin [168]. In vitro studies revealed that IL-11 increased chemoresistance through gp130/JAK/STAT3/BCL-2-mediated anti-apoptosis signaling pathway [169].

Other cytokines, such as IL-33, prevented drug-induced apoptosis after treatment with platinum derivatives by activating the JNK signaling pathway in GAC cells [170], while A proliferation-inducing ligand (APRIL) and fibroblast growth factor-inducible-14 (Fn14), both members of the tumor necrosis factor (TNF) family, are involved in GAC resistance to cisplatin and 5-FU, respectively, via NF-κB activation [164,166].

GAC cells can secrete autocrine cytokines, such as CCL2, able to induce and maintain cisplatin resistance by inactivating proapoptotic autophagy via PI3K-AKT-mTOR signaling [165]. Autophagy modulation by miRNAs has been associated with chemoresistance. Thus, overexpression of miR-23b-3p reversed resistance to both 5-FU and cisplatin mediated by autophagy-related gene-12 (*ATG-12*) and high-mobility group box 2 (*HMGB2*) [177]. In the same sense, ATG-5 up-regulation and the subsequent autophagy activation were associated with shorter OS in GAC patients receiving epirubicin, cisplatin, and 5-FU adjuvant chemotherapy after surgical resection [171].

The interaction of exosomes with the GAC microenvironment can also contribute to the development of drug resistance. Mesenchymal stem cell (MSC)-derived exosomes induce resistance to 5-FU by up-regulating ABC pumps and by inhibiting apoptosis of GAC cells [175], while tumor-associated macrophage (TAM)-derived exosomes induce cisplatin resistance in GAC cells by transferring miR-21a-5p, which inhibits apoptosis and activates PI3K/AKT pathway [176].

GAC cells can thrive in an unfavorable microenvironment by increasing glycolysis rate and decreasing mitochondrial function (Warburg effect), which can contribute to drug resistance. In fact, enhanced expression of glycolysis-associated enzymes has been correlated with hypoxia-induced 5-FU resistance in vitro [173]. Another study reported that hypoglycemia affected the PI3K/mTOR pathway and increased resistance to 5-FU and other drugs, especially in GAC cells dependent on glycolysis [172]. In addition, MSC-derived lncRNA HCP5 increases fatty acid oxidation, promotes stemness, and enhances resistance to oxaliplatin and 5-FU in GAC cells [174].

## 8. Mechanisms of Chemoresistance Type 7 (MOC-7)

Phenotypical changes associated with the appearance of mesenchymal and stem cell features result in a reduced response of GAC to chemotherapy. During aberrant EMT, cancer cell polarity and adhesion are impaired, increasing their migratory behavior, invasiveness, and resistance to apoptosis [178] (Table 7). In GAC, EMT is triggered by extracellular signals from the tumor microenvironment, such as transforming growth factor-β (TGF-β), HGF, and HIF-1α (MOC-6), and intracellular processes, such as the overactivation of survival pathways (MOC-5) [178].

The TGF-β signaling pathway is involved in many cellular processes, including cell growth, differentiation, and apoptosis, and plays a crucial role in EMT promotion and chemoresistance in GAC [179]. There is a wide variety of receptors and ligands involved in the activation of this pathway. In this sense, the up-regulation of CD168, also known as hyaluronan-mediated motility receptor (HMMR), has been associated with the TGF-β-mediated induction of EMT markers, such as vimentin and N-cadherin, and a worse response of GAC to 5-FU [179]. Moreover, the crosstalk between miRNAs and TGF-β can regulate EMT-mediated chemoresistance in GAC. Consistently, miRNA-mediated inhibition of the receptor TGFBR2 sensitizes 5-FU-resistant GAC cells [180]. In these cells, miR-577 is up-regulated, which has been associated with an unfavorable prognosis [181]. Besides, miR-577 enhances the TGF-β pathway by targeting the serum deprivation protein response (SDPR), which induces EMT resulting in increased resistance to oxaliplatin [181]. Furthermore, miR-187 down-regulation decreases the sensitivity of GAC cells to cisplatin by up-regulating the DNA repair enzymes ERCC1/4 (MOC-4) and increasing the activity of the TGF-β/SMAD4 pathway [182].

Cancer stem cells (CSCs) in GAC share some phenotypic traits, such as chemoresistance, with cells undergoing EMT [183]. These cells can originate from the bone marrow or the stomach itself by oncogenic mutations in progenitor cells [184]. Gastric CSCs and cells undergoing EMT are heterogeneous regarding their genetic signature and phenotype. Each cell subtype is characterized by a pattern of protein expression, including cell adhesion surface glycoproteins (CD44, CD24, CD90, CD133, CXCR4, and EpCAM), enzymes (aldehyde dehydrogenase 1 or ALDH1), and transcription factors (SOX2, SNAIL1, STAT3, TWIST1, ZEB1, and ZEB2) [184].

CD44, a characteristic marker of CSCs, is a cell surface adhesion molecule expressed in a variety of epithelial cells and stem cells. In combination with the EMT markers SNAIL1 and vimentin, CD44 has been suggested as prognostic biomarker in GAC [185]. CD44 overexpression results in higher activity of the Hedgehog survival pathway [142]. Aberrant alternative splicing produces CD44 isoforms that are overexpressed in carcinomas, including GAC, whereas the standard CD44 isoform is predominantly expressed in normal cells. In addition, the pattern of appearance of these splicing forms in GAC is different in intestinal-type tumors, diffuse-type tumors, and even in early-stage tumors [186]. Proteins resulting from CD44 splicing forms act as co-receptors of c-Met, HGF, VEGF, and Hedgehog signaling pathways to activate cell proliferation [186]. Thus, CD44 and its variants are not simply CSCs markers of GAC but are also actively involved in the initiation and progression of the disease. Moreover, it has been shown that CD44^+^ GAC cells are markedly resistant to cell death induced by 5-FU and etoposide [183]. Also, GAC patients with high CD44 expression who were treated with the FOLFOX regimen had a lower OS [142]. Not only CD44 has been identified as a typical CSC marker individually but also in combination with other markers, such as CD24, CD133, and EpCAM, to characterize CSCs in GAC. Thus, CD44^+^/EpCAM^+^ cells isolated from GAC exhibited enhanced resistance to 5-FU, anthracyclines, and taxanes [187]. When the up-regulation of CD44 is accompanied by CD24 absence, the OS of patients treated with 5-FU was shorter [188]. However, CD24 expression favors migration, invasiveness, and acquired chemoresistance under hypoxic conditions, such as after long-term 5-FU treatment [189].

CD133 (*PROM1* gene) is a transmembrane glycoprotein widely distributed in the body, whose function is probably to organize the apical plasma membrane in epithelial cells. CD133 is a recognized marker of CSCs in many cancers, including GAC. High CD133 expression in GAC has been associated with chemoresistance since patients with CD133^+^ tumors treated with an adjuvant cisplatin/5-FU regimen had shorter OS and DFS than those with CD133^-^ tumors [190]. Interestingly, CD133 induces the up-regulation of MDR1 and BCL-2 through PI3K/AKT pathway activation [191]. Although the expression of the CSC-associated glycoproteins mentioned above has been related to GAC refractoriness to chemotherapy, subtypes of CSCs that express CD90 (thymocyte differentiation antigen 1) respond better to some drugs like trastuzumab [192].

Diffuse-type GAC is characterized by extensive stromal fibrosis, poor vascularization, considerable chemoresistance, and the presence of quiescent CSCs. These tumors show increased TGF-β activity and expression of CXCR4, a marker of CSC highly resistant to docetaxel [194].

ALDH1A3 and ALDH1L1 are two ALDH1 isoenzymes involved in protecting against the toxic effects of reactive oxygen species. Their high expression in CSCs correlated with a worse OS in GAC patients treated with 5-FU [195].

LGR5, a member of the Wnt/β-catenin pathway closely related to EMT signature, is a therapeutic target and a prognostic biomarker in GAC [200]. LGR5 overexpression has been associated with shorter OS in patients treated with FOLFOX [196]. LGR5 can up-regulate EMT inducers (PRRX1) and stemness genes, such as *SOX2*, *OCT4*, and *NANOGP8*, in GAC cells [200]. Indeed, in GAC cells, the expression of *NANOGP8* activates Wnt/β-catenin leading to enhanced oxaliplatin resistance [198].

Doublecortin-like kinase 1 (*DCLK1*) is a transmembrane microtubule-related kinase involved in the promotion of stemness and EMT markers, such as SOX2, OCT4, SLUG, and SNAIL among others, in many solid tumors [201]. Indeed, it has been suggested as a specific marker of gastric CSCs [202] and overexpression of *DCLK1* can induce EMT in GAC cell lines through Notch activation [203]. An analysis using RNA sequencing data from “The Cancer Genome Atlas” (TCGA) showed that high *DCLK1* expression predicts worse OS and PFS in patients with GAC and is linked with functional regulation of the tumor microenvironment (MOC-6) [204]. DCLK1 can confer resistance to drugs used in GAC treatment, e.g., cisplatin and 5-FU [205,206]. However, the role of DCLK1 in GAC chemoresistance has not been studied yet.

MOC-7 can also develop during long-term treatment with anti-cancer drugs. For example, oxaliplatin and doxorubicin can induce EMT in GAC cells through Fas and β-catenin signaling, respectively [207,208]. After long-term exposure of GAC to trastuzumab, acquired resistance to this drug can be developed as a result of EMT activation through the TGF-β-miR-200c-ZEB2 axis [197]. Continuous exposure in vitro of GAC cells to trastuzumab can also up-regulate the human epidermal growth factor receptor 4 (HER4) and induce EMT by activating YAP1-PI3K signaling, which promotes resistance to this drug [148].

The absence of the transferrin receptor CD71 characterizes a subpopulation of CSCs in GAC with elevated resistance to 5-FU. The proportion of these CSCs in the tumor increases during treatment with 5-FU [193]. Moreover, 5-FU-based chemotherapy favors the enrichment of the tumor with side-population cells, a subtype of CSCs with high expression of BCRP and MDR1 that are strongly resistant to 5-FU, through Hedgehog activation [31]. SOX2, which is a transcription factor that up-regulates *ABCG2*, is also highly overexpressed in side-population cells, conferring resistance to cisplatin and doxorubicin [199].

## 9. Conclusions and Perspectives

Despite the lacking/poor response of GAC to classical or vectorized pharmacological treatment, this is the only hope for many patients with advanced GAC who are not eligible for undergoing surgical removal of the tumor. The possibility of improving this landscape necessarily requires a better understanding of the molecular and cellular mechanisms underlying GAC chemoresistance. The current knowledge in the field of oncological pharmacology regarding GAC has been revised here, highlighting the marked complexity of the problem, as different mechanisms can be expressed at the same type in the tumor cell contributing to an impaired response to several anticancer agents. The available information should be the starting point for carrying out further investigations aimed at developing novel drugs and pharmacological strategies to overcome GAC chemoresistance. These might include the enhancement of drug uptake by increasing the activity or expression of SLC transporters or synthesizing new analogs, more selectively vectorized to these transporters. Alternatively, enhanced tumor targeting could be also achieved by drug encapsulation into a diverse panel of nanoparticles, as it has been investigated in GAC and other tumors [209]. Another alternative for enhancing GAC sensitivity is the reduction of drug efflux through manipulation of the interaction between anticancer drugs and export pumps [9]. Further possibilities, whose usefulness may be shared by many cancers, include those aimed at altering the balance between apoptosis and survival, or taking advantage of the collateral sensitivity occurring in cancer cells in response to treatment. These promising perspectives should be thoroughly considered in future research. On the other hand, further research regarding the molecular basis of chemoresistance will also allow to identify novel biomarkers that predict the responsiveness of GAC patients to certain drugs, which could help personalized medicine to choose the best pharmacological treatment for each patient.

## Figures and Tables

**Table 1:** Mechanisms of chemoresistance type 1 (MOC-1) to drugs used in the treatment of GAC.

Protein	Feature	Drugs Affected	Consequences	Ref.
Uptake carriers (MOC-1a)				
CTR1	Down-regulation	Cisplatin	Decreased sensitivity	[9,10]
OATP1B3	Alternative TSS	Irinotecan, Docetaxel, Methotrexate	Unknown	[9]
Export pumps (MOC-1b)				
MDR1	Up-regulation *	Platinum derivatives,	Reduced clinical response	[14]
		5-FU, Epirubicin	Decreased cell sensitivity in vitro	[17,18]
MRP1	Up-regulation	Cisplatin, Doxorubicin	Decreased cell sensitivity in vitro	[22,23,24,25]
MRP2	GV (rs717620; CC)	5-FU, Oxaliplatin	Worse response	[26]
MRP4	Up-regulation	Cisplatin, Dasatinib,	Decreased cell sensitivity in vitro	[27,28]
		5-FU	Increased risk of cancer relapse	[31]
BCRP	Up-regulation	Cisplatin	Reduced OS	[30]
ATP7A	Up-regulation	Oxaliplatin	Decreased cell sensitivity in vitro	[33]
ATP7B	Up-regulation	Cisplatin	Decreased cell sensitivity in vitro	[34]

5-FU, 5-Fluorouracil; GV, genetic variant; OS, overall survival; TSS, transcription start site; *, Contradictory data.

**Table 2:** Mechanisms of chemoresistance type 2 (MOC-2) to drugs used in the treatment of GAC.

Protein	Feature	Drugs affected	Consequences	Ref.
CYP2A6	Inactivating GVs	Tegafur (5-FU)	Reduced DFS and OS	[37,38,46]
DPD	Up-regulation	5-FU	Reduced OS	[47,48]
GST-pi	Up-regulation	5-FU, Cisplatin, Mitomycin C	Increased resistance in vitro	[49,50]
MTs	Up-regulation *	Cisplatin, Irinotecan (SN-38)	Reduced clinical response *	[51,52]
TP	Down-regulation *	5-FU	Reduced OS *	[41,42]

5-FU, 5-Fluorouracil; DFS, disease-free survival; GV, genetic variant; OS, overall survival; *, Contradictory data.

**Table 3:** Mechanisms of chemoresistance type 3 (MOC-3) to drugs used in the treatment of GAC.

Protein	Feature	Drug Affected	Consequences	Ref.
HER2	Low expression	Trastuzumab	Reduced OS	[78,79]
TS	High expression	Capecitabine, Oxaliplatin	Worse outcome *	[67]
TS	GV: 2R/2R or 2R/3R	Cisplatin, Oxaliplatin, 5-FU	Reduced OS	[68]
TUBB3	High expression	Taxanes, Cisplatin, 5-FU	Worse clinical outcome	[66,70,71,72]
VEGF	Low expression	Bevacizumab	Worse clinical outcome	[85]
VEGFR-2	High expression	Ramucirumab	Shorter PFS	[81]

5-FU, 5-fluorouracil; GV, genetic variant; OS, overall survival; PFS, progression-free survival; *, Contradictory data.

**Table 4:** Mechanisms of chemoresistance type 4 (MOC-4) to drugs used in the treatment of GAC.

Factor	Feature	Drugs Affected	Consequences	Ref.
Nucleotide-excision DNA repair (NER)				
ERCC1	High expression	FOLFOX	Reduced OS and TTP	[65]
ERCC1	High expression	Platinum derivatives	Reduced OS and response	[88]
ERCC1	GV:rs11615	5-FU, Oxaliplatin	Reduced OS and response *	[92,93]
ERCC1	GV:rs3212986	Cisplatin, FOLFOX	Worse clinical outcome *	[94,96]
ERCC2	Up-regulation	FOLFOX	Reduced OS, PFS and response	[98]
ERCC4	High expression	Cisplatin	Decreased sensitivity in vitro	[91,99]
Base-excision DNA repair (BER)				
XRCC1	Up-regulation	Cisplatin	Increased drug resistance in vitro	[100]
XRCC1	GV:rs25487	Oxaliplatin	Worse clinical outcome	[101]
Mismatch repair (MMR)				
MLH1	Lack of expression	5-FU	Reduced response	[102]
MSI	Appearance	5-FU	Reduced DFS	[103]

5-FU, 5-fluorouracil; DFS, disease-free survival; FOLFOX, (leucovorin/5-FU/oxaliplatin); GV, genetic variant; MSI, microsatellite instability; OS, overall survival; PFS, progression-free survival; TTP, time-to-progression; *, Contradictory data.

**Table 5:** Mechanisms of chemoresistance type 5 (MOC-5) to drugs used in the treatment of GAC.

Factor	Feature	Drugs Affected	Consequences	Ref.
Pro-apoptotic factors (MOC-5a)				
BAK	Down-regulation	Docetaxel	Decreased sensitivity in vitro	[126]
BAX	Down-regulation	5-FU, Capecitabine, Cisplatin, Irinotecan, Oxaliplatin	Reduced OS and PFS	[123,124,125]
BIM	Down-regulation	Docetaxel	Reduced OS	[127]
miR-501	Up-regulation	Doxorubicin	Decreased sensitivity in vitro	[129]
miR-633	Up-regulation	Doxorubicin	Decreased sensitivity in vitro and in vivo	[132]
miR-BART20-5p	Up-regulation	5-FU, Docetaxel	Decreased sensitivity in vitro	[128]
p16^INKa^	Down-regulation	5-FU	Reduced response *	[120,121]
p53	Down-regulation	5-FU, Cisplatin, Etoposide, Mitomycin C	Reduced response	[137,138]
p53	Loss of activity	5-FU, Cisplatin, Paclitaxel	Reduced OS	[111,112]
p53	Gain-of-function GV	First-line chemotherapy	Reduced OS and PFS	[116,117]
Survival pathways (MOC-5b)				
β-catenin, APC, FBXW7	Mutations	First-line chemotherapy	Reduced OS and PFS	[139]
COX-2	Up-regulation	Oxaliplatin, Irinotecan	Decreased sensitivity in vitro	[140]
E-cadherin	Down-regulation	5-FU, Cisplatin	Reduced DFS	[141]
GLI1, GLI2	Up-regulation	5-FU	Reduced clinical response	[31]
Hedgehog	Increased activity	5-FU, Cisplatin	Reduced OS	[142]
JAK/STAT3	Increased activity	Cisplatin	Reduced OS	[143]
NFκB	Increased activity	5-FU, Capecitabine, Cisplatin	Decreased sensitivity in vitro	[14,134,135]
Notch 1	Up-regulation	5-FU, Cisplatin	Reduced OS	[144]
PI3K/AKT	Increased activity	Trastuzumab	Reduced OS and PFS	[145]
SHH, GLI1	Up-regulation	Doxorubicin	Decreased sensitivity in vitro	[146]
Survivin	Up-regulation	5-FU, Cisplatin, Docetaxel	Increased DPR	[136]
WNT/β-catenin	Increased activity	Cisplatin	Reduced OS and DFS	[147]
YAP1	Up-regulation	Trastuzumab	Decreased sensitivity in vitro	[148]
YAP1, TAZ	Up-regulation	5-FU, Cisplatin	Reduced OS and PFS	[139]

5-FU, 5-fluorouracil; DFS, disease-free survival; DPR, disease progression rate; GV, genetic variant; OS, overall survival; PFS, progression-free survival; *, Contradictory data.

**Table 6:** Mechanisms of chemoresistance type 6 (MOC-6) to drugs used in the treatment of GAC.

Factor	Feature	Drugs Affected	Consequences	Ref.
Hypoxia				
HIF-1α	Up-regulation	5-FU, Platinum derivatives	Apoptosis inhibition	[156,157,158,159]
	Up-regulation	5-FU	Relapse after treatment	[161]
STC1	Up-regulation	Cisplatin	Apoptosis inhibition	[162]
Immune system and inflammation				
APRIL	Increased production	Cisplatin	Apoptosis inhibition	[164]
CCL2	Increased production	Cisplatin	Apoptosis inhibition	[165]
Fn14	Increased production	5-FU	Apoptosis inhibition	[166]
IL-6	Increased production	5-FU	Poor response	[167]
IL-8	Increased production	Platinum derivatives	*ABCB1* overexpression and Apoptosis inhibition	[14,168]
IL-11	Increased production	Several drugs	Apoptosis inhibition	[169]
IL-33	Increased production	Platinum derivatives	Apoptosis inhibition	[170]
NR4A2	High expression	5-FU	Apoptosis inhibition and worse survival rates	[163]
Others				
ATG-5	High expression	5-FU, Cisplatin, Epirubicin	Poor survival	[171]
Glycemia	Low levels	5-FU	Metabolic reprogramming and activation of survival	[172]
Glycolysis enzymes	Up-regulation	5-FU	Metabolic reprogramming	[173]
lncRNA HCP5	Production	5-FU, Oxaliplatin	Metabolic reprogramming	[174]
MSC-Exosomes	Production	5-FU	Activation of other MOCs	[175]
TAM-Exosomes	miR-21a-5p transfer	Cisplatin	Apoptosis inhibition	[176]

5-FU, 5-fluorouracil; MOCs, mechanisms of chemoresistance.

**Table 7:** Mechanisms of chemoresistance type 7 (MOC-7) to drugs used in the treatment of GAC.

Factor	Feature	Drugs Affected	Consequences	Ref.
*Cell adhesion proteins*				
CD133	Up-regulation	5-FU, Cisplatin	Reduced OS and DFS	[190]
CD44	Up-regulation	5-FU, Etoposide	Decreased sensitivity in vitro	[183]
CD44	Up-regulation	5-FU, Oxaliplatin	Decreased clinical response	[142]
CD44/CD24	Up-/Down-regulation	5-FU	Reduced OS	[188]
CD44/EpCAM	Up-regulation	5-FU, Doxorubicin, Paclitaxel	Decreased sensitivity in vitro	[187]
CD71	Down-regulation	5-FU	Decreased sensitivity in vitro and in vivo	[193]
CXCR4	Up-regulation	Docetaxel	Decreased sensitivity in vitro	[194]
*Enzymes*				
ALDH1	Up-regulation	5-FU	Reduced OS	[195]
*Survival pathways*				
Hedgehog	Increased activity	5-FU	Decreased sensitivity in vitro	[31]
HER4	Up-regulation	Trastuzumab	Decreased sensitivity in vitro and in vivo	[148]
HMMR	Up-regulation	5-FU	Reduced OS	[179]
LGR5	Up-regulation	5-FU, Oxaliplatin	Reduced OS	[196]
miR-187	Down-regulation	Cisplatin	Decreased sensitivity in vitro	[182]
miR-577	Up-regulation	Oxaliplatin	Decreased sensitivity in vitro	[181]
TGF-β/ZEB2	Increased activity	Trastuzumab	Decreased sensitivity in vitro	[197]
TGFBR2	Up-regulation	5-FU	Decreased sensitivity in vitro	[180]
*Transcription factors*				
NANOGP8	Up-regulation	Oxaliplatin	Decreased sensitivity in vitro	[198]
SOX2	Up-regulation	Cisplatin, Doxorubicin	Decreased sensitivity in vitro and in vivo	[199]

5-FU, 5-fluorouracil; DFS, disease-free survival; OS, overall survival.

## Abbreviations

5-FU, 5-fluorouracil; ABC, ATP-binding cassette; AKT, α-serine/threonine-protein kinase; ALDH, aldehyde dehydrogenase; APC, Adenomatous polyposis coli; APRIL, A proliferation-inducing ligand; ATG, autophagy-related gene; ATP7A, ATPase copper transporting α; BAD, Bcl2 antagonist of cell death; BAK, Bcl2 antagonist/killer; BAX, Bcl2 associated X protein; BCL-XL, apoptosis regulator BCL, long isoform; BCRP, breast cancer resistance protein; BER, base excision repair; BID, BH3 interacting domain death agonist; BIM, Bcl-2 interacting mediator of cell death; BLID, BH3-like motif-containing cell death inducer; BRCA, breast cancer susceptibility protein; CAF, cancer-associated fibroblast; CCL2, C-C motif chemokine ligand 2; CES, carboxylesterase 2; COX-2, cyclooxygenase-2; CSC, cancer stem cell; CTNNB1, catenin β 1; CTR1, copper transport protein; CXCR4, C-X-C motif chemokine receptor 4; CYP, cytochrome; DCF, Docetaxel/Cisplatin/Fluorouracil; DCTPP1, dCTP pyrophosphatase 1; CDDP, *cis*-diamminedichloroplatinum or cisplatin; DFS, disease-free survival; DPD, dihydropyrimidine dehydrogenase; DPR, disease progression rate; EGFR, epidermal growth factor receptor; EMT, epithelial-mesenchymal transition; ENT, equilibrative nucleoside transporter; ERCC1/2, excision repair cross-complementation 1/2; FADD, Fas associated via death domain; FBXW7, F-Box and WD-40 domain-containing protein 7; Fn-14, fibroblast growth factor-inducible-14; FOLFOX, oxaliplatin/leucovorin/5-FU regimen; GAC, gastric adenocarcinoma; GLI, glioma-associated oncogene homolog; GST, glutathione-S-transferase; GV, genetic variant; HER2, human epidermal growth factor receptor 2; HIF, hypoxia-inducible transcription factor; HMG, high-mobility group box; HMMR, hyaluronan-mediated motility receptor; HR, homologous recombination; IL, interleukin; lncRNA, long non-coding RNA; JAK, Janus kinase 2; MAPT, microtubule-associated protein tau; mDCF, modified Docetaxel-Cisplatin-Fluorouracil; MDR, multidrug resistance; MMR, mismatch repair system; MOC, mechanism of chemoresistance; MRP, multidrug resistance-associated protein; MSC, mesenchymal stem cell; MSI, microsatellite instability; MT, metallothionein; NANOG, homeobox transcription factor Nanog; NER, nucleotide excision repair; NF-ĸB, nuclear factor κB; NR4A2, orphan nuclear receptor 4A2; OATP, organic anion transporting polypeptide; OCT4; octamer-binding transcription factor 4; OS, overall survival; PFS, progression-free survival; PI3K, phosphoinositide 3-kinase; SHH, sonic hedgehog; SLC, solute carrier; SMAD, mothers against decapentaplegic homolog; SNP, single nucleotide polymorphism; SOX, SRY-box transcription factor; SP, side-population; STAT3, signal transducer and activator of transcription 3; STC-1, stanniocalcin-1; TAM, tumor-associated macrophage; TAZ, transcriptional coactivator with PDZ-binding motif; TKI, tyrosine kinase inhibitor; TNF, tumor necrosis factor; TOPO, DNA topoisomerase; TP, thymidine phosphorylase; TP53, tumor protein 53; TS, thymidylate synthase; TTP, time-to-progression; TUBB3, β-tubulin-III; UDP, UDP-glucuronosyl transferase; UTR, untranslated region; VEGF, vascular endothelial growth factor; VEGFR 2, vascular endothelial growth factor receptor 2; WNT, wingless integration site; XIAP, X-linked inhibitor of apoptosis protein; XRCC1, X-ray repair cross complementing 1; YAP1, yes-associated protein 1; ZEB2, zinc finger E-box-binding homeobox 2.
